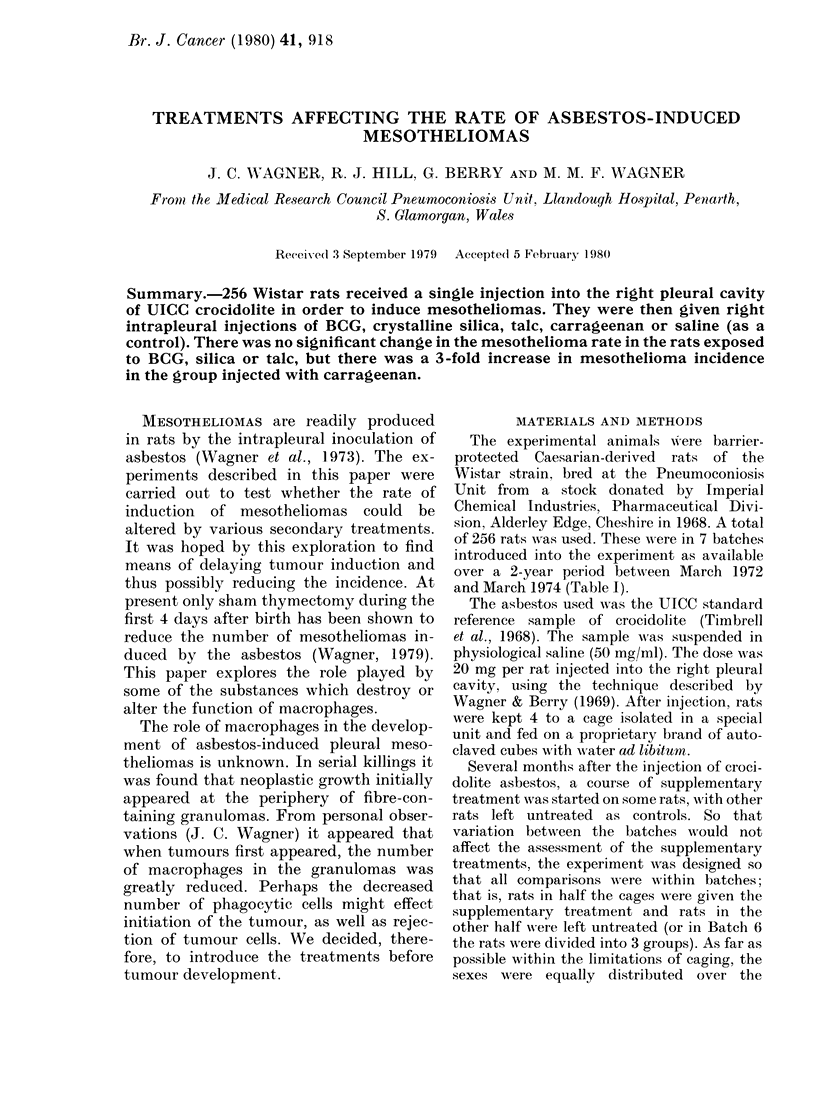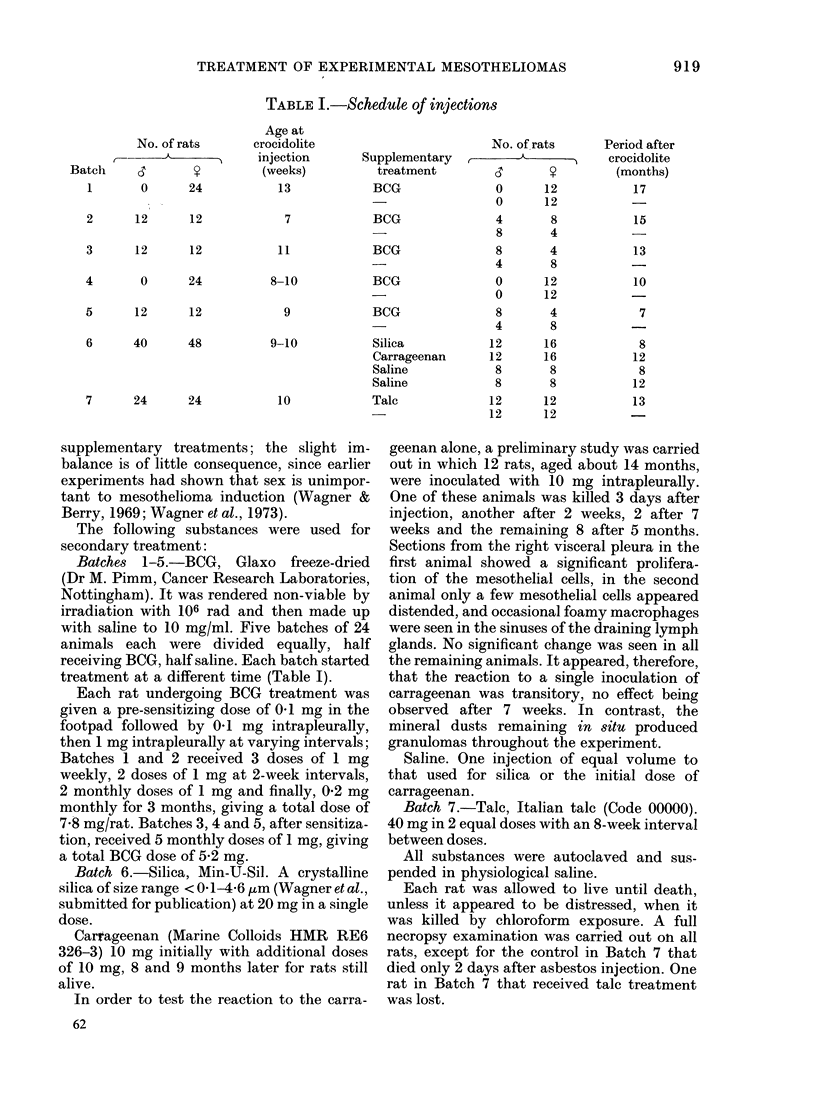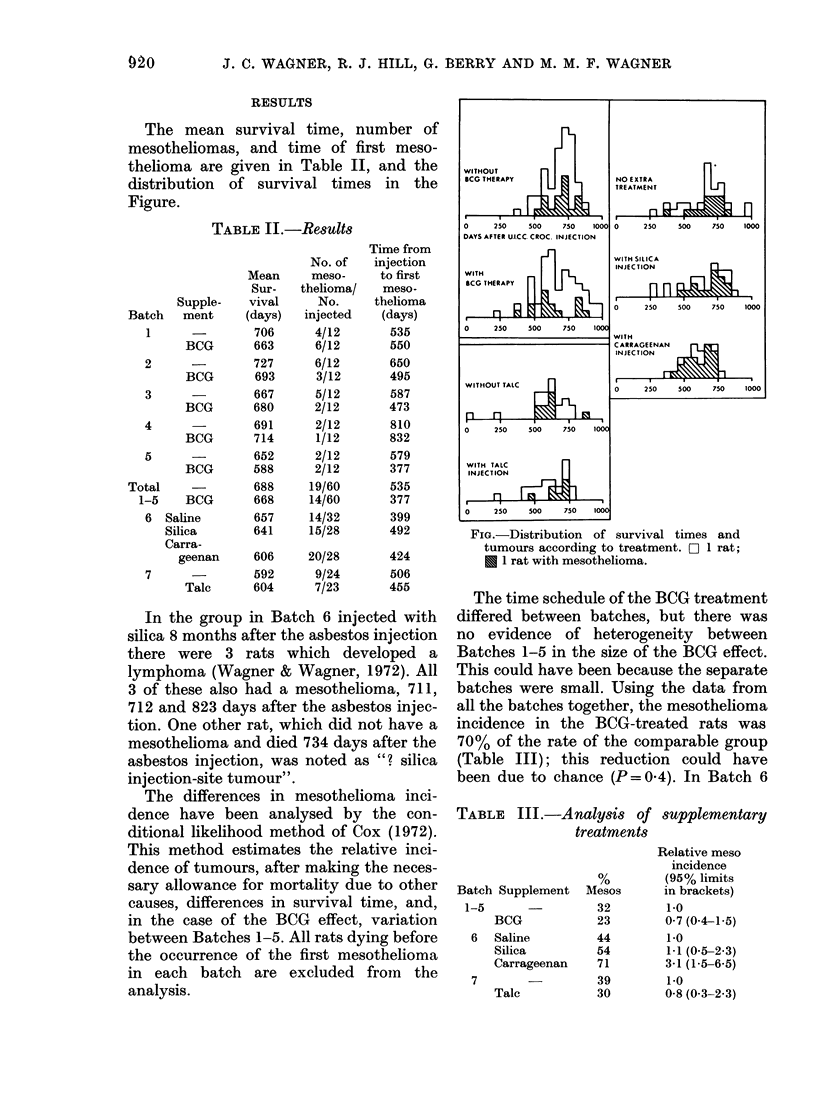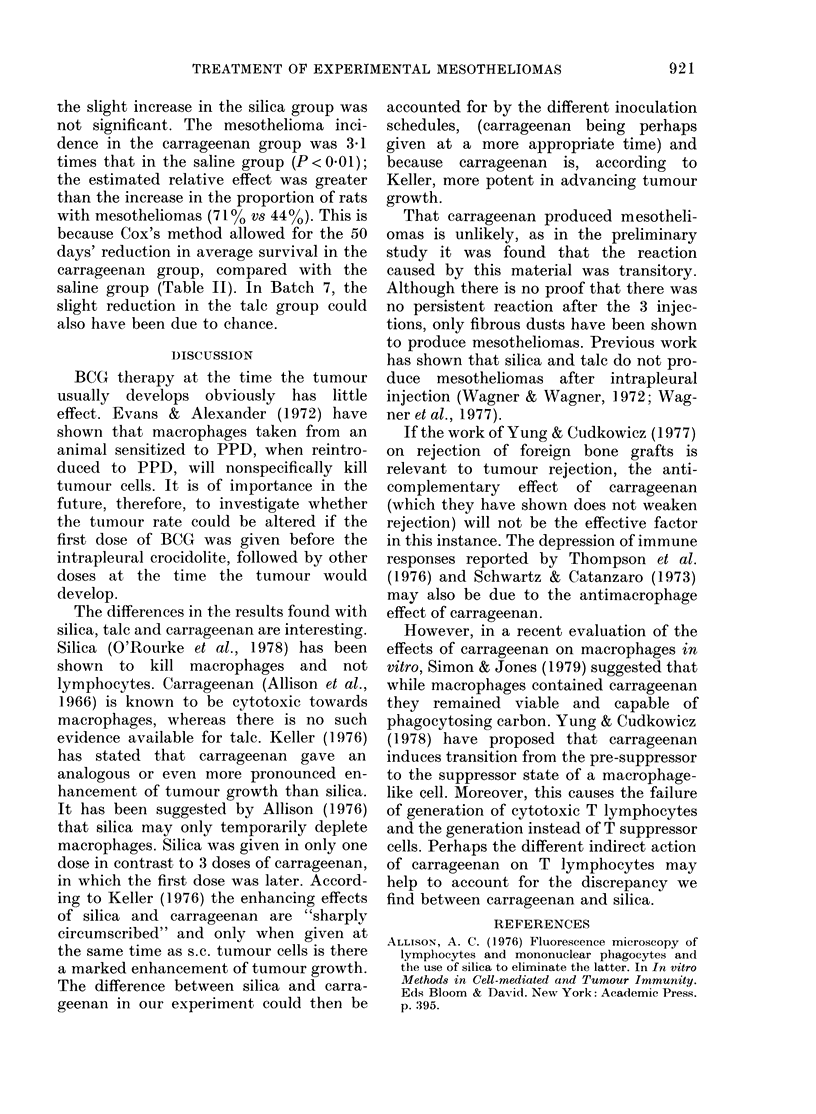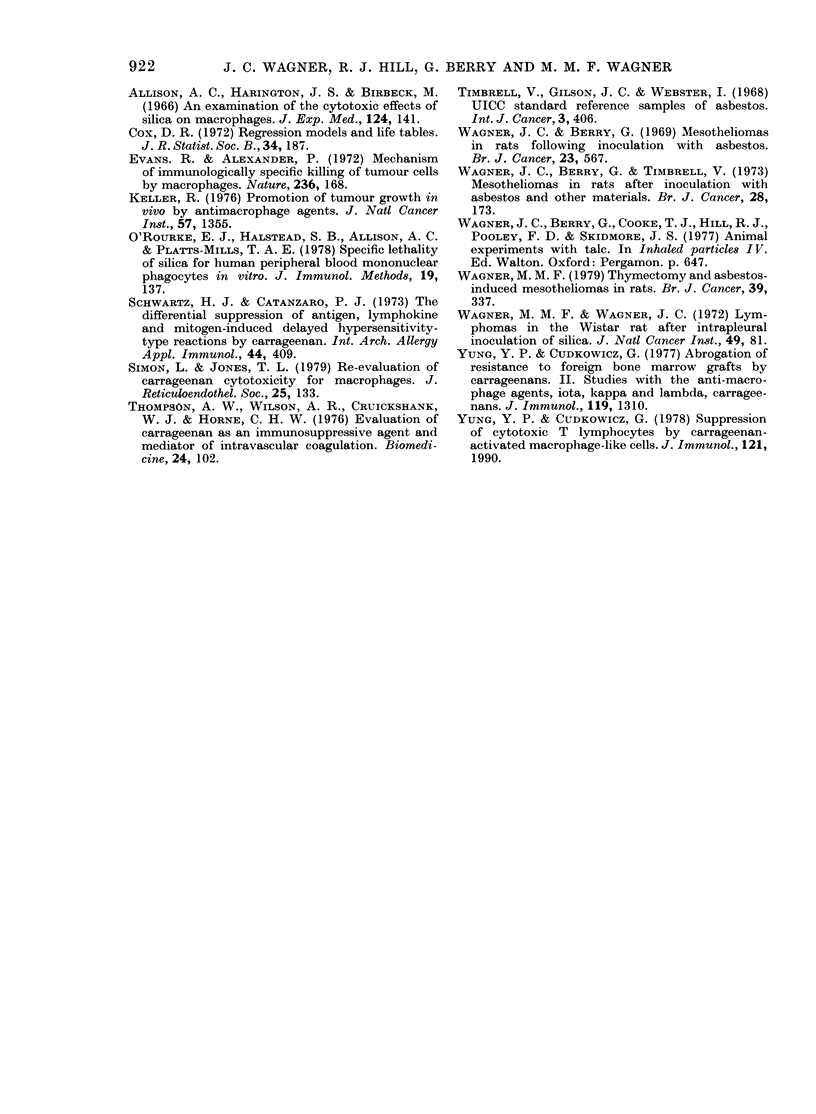# Treatments affecting the rate of asbestos-induced mesotheliomas.

**DOI:** 10.1038/bjc.1980.169

**Published:** 1980-06

**Authors:** J. C. Wagner, R. J. Hill, G. Berry, M. M. Wagner

## Abstract

256 Wistar rats received a single injection into the right pleural cavity of UICC crocidolite in order to induce mesotheliomas. They were then given right intrapleural injectons of BCG, crystalline silica, talc, carrageenan or saline (as a control). There was no significant change in the mesothelioma rate in the rats exposed to BCG, silica or talc, but there was a 3-fold increase in mesothelioma incidence in the group injected with carrageenan.


*Br. J. Cancer* (1980) **41**, 918

### TREATMENTS AFFECTING THE RATE OF ASBESTOS-INDUCED MESOTHELIOMAS

J. C. WAGNER, R. J. HILL, G. BERRY AND M. M. F. WAGNER

*From the Medical Research Council Pneumoconiosis Unit, Llandough Hospital, Penarth, S. Glamorgan, Wales*

Received 3 September 1979 Accepted 5 February 1980

**Summary.—256 Wistar rats received a single injection into the right pleural cavity of UICC crocidolite in order to induce mesotheliomas. They were then given right intrapleural injections of BCG, crystalline silica, talc, carrageenan or saline (as a control). There was no significant change in the mesothelioma rate in the rats exposed to BCG, silica or talc, but there was a 3-fold increase in mesothelioma incidence in the group injected with carrageenan.**

MESOTHELIOMAS are readily produced in rats by the intrapleural inoculation of asbestos (Wagner *et al.*, 1973). The experiments described in this paper were carried out to test whether the rate of induction of mesotheliomas could be altered by various secondary treatments. It was hoped by this exploration to find means of delaying tumour induction and thus possibly reducing the incidence. At present only sham thymectomy during the first 4 days after birth has been shown to reduce the number of mesotheliomas induced by the asbestos (Wagner, 1979). This paper explores the role played by some of the substances which destroy or alter the function of macrophages.

The role of macrophages in the development of asbestos-induced pleural mesotheliomas is unknown. In serial killings it was found that neoplastic growth initially appeared at the periphery of fibre-containing granulomas. From personal observations (J. C. Wagner) it appeared that when tumours first appeared, the number of macrophages in the granulomas was greatly reduced. Perhaps the decreased number of phagocytic cells might effect initiation of the tumour, as well as rejection of tumour cells. We decided, therefore, to introduce the treatments before tumour development.

### MATERIALS AND METHODS

The experimental animals were barrier-protected Caesarian-derived rats of the Wistar strain, bred at the Pneumoconiosis Unit from a stock donated by Imperial Chemical Industries, Pharmaceutical Division, Alderley Edge, Cheshire in 1968. A total of 256 rats was used. These were in 7 batches introduced into the experiment as available over a 2-year period between March 1972 and March 1974 (Table I).

The asbestos used was the UICC standard reference sample of crocidolite (Timbrell *et al.*, 1968). The sample was suspended in physiological saline (50 mg/ml). The dose was 20 mg per rat injected into the right pleural cavity, using the technique described by Wagner & Berry (1969). After injection, rats were kept 4 to a cage isolated in a special unit and fed on a proprietary brand of autoclaved cubes with water *ad libitum*.

Several months after the injection of crocidolite asbestos, a course of supplementary treatment was started on some rats, with other rats left untreated as controls. So that variation between the batches would not affect the assessment of the supplementary treatments, the experiment was designed so that all comparisons were within batches; that is, rats in half the cages were given the supplementary treatment and rats in the other half were left untreated (or in Batch 6 the rats were divided into 3 groups). As far as possible within the limitations of caging, the sexes were equally distributed over the

TABLE I.—*Schedule of injections*

| Batch | No. of rats ♂ | No. of rats ♀ | Age at crocidolite injection (weeks) | Supplementary treatment | No. of rats ♂ | No. of rats ♀ | Period after crocidolite (months) |
|---|---|---|---|---|---|---|---|
| 1 | 0 | 24 | 13 | BCG | 0 | 12 | 17 |
| | | | | — | 0 | 12 | — |
| 2 | 12 | 12 | 7 | BCG | 4 | 8 | 15 |
| | | | | — | 8 | 4 | — |
| 3 | 12 | 12 | 11 | BCG | 8 | 4 | 13 |
| | | | | — | 4 | 8 | — |
| 4 | 0 | 24 | 8–10 | BCG | 0 | 12 | 10 |
| | | | | — | 0 | 12 | — |
| 5 | 12 | 12 | 9 | BCG | 8 | 4 | 7 |
| | | | | — | 4 | 8 | — |
| 6 | 40 | 48 | 9–10 | Silica | 12 | 16 | 8 |
| | | | | Carrageenan | 12 | 16 | 12 |
| | | | | Saline | 8 | 8 | 8 |
| | | | | Saline | 8 | 8 | 12 |
| 7 | 24 | 24 | 10 | Talc | 12 | 12 | 13 |
| | | | | — | 12 | 12 | — |

supplementary treatments; the slight imbalance is of little consequence, since earlier experiments had shown that sex is unimportant to mesothelioma induction (Wagner & Berry, 1969; Wagner *et al.*, 1973).

The following substances were used for secondary treatment:

*Batches* 1–5.—BCG, Glaxo freeze-dried (Dr M. Pimm, Cancer Research Laboratories, Nottingham). It was rendered non-viable by irradiation with $10^6$ rad and then made up with saline to 10 mg/ml. Five batches of 24 animals each were divided equally, half receiving BCG, half saline. Each batch started treatment at a different time (Table I).

Each rat undergoing BCG treatment was given a pre-sensitizing dose of 0·1 mg in the footpad followed by 0·1 mg intrapleurally, then 1 mg intrapleurally at varying intervals; Batches 1 and 2 received 3 doses of 1 mg weekly, 2 doses of 1 mg at 2-week intervals, 2 monthly doses of 1 mg and finally, 0·2 mg monthly for 3 months, giving a total dose of 7·8 mg/rat. Batches 3, 4 and 5, after sensitization, received 5 monthly doses of 1 mg, giving a total BCG dose of 5·2 mg.

*Batch* 6.—Silica, Min-U-Sil. A crystalline silica of size range $<0{\cdot}1$–$4{\cdot}6\ \mu m$ (Wagner *et al.*, submitted for publication) at 20 mg in a single dose.

Carrageenan (Marine Colloids HMR RE6 326–3) 10 mg initially with additional doses of 10 mg, 8 and 9 months later for rats still alive.

In order to test the reaction to the carrageenan alone, a preliminary study was carried out in which 12 rats, aged about 14 months, were inoculated with 10 mg intrapleurally. One of these animals was killed 3 days after injection, another after 2 weeks, 2 after 7 weeks and the remaining 8 after 5 months. Sections from the right visceral pleura in the first animal showed a significant proliferation of the mesothelial cells, in the second animal only a few mesothelial cells appeared distended, and occasional foamy macrophages were seen in the sinuses of the draining lymph glands. No significant change was seen in all the remaining animals. It appeared, therefore, that the reaction to a single inoculation of carrageenan was transitory, no effect being observed after 7 weeks. In contrast, the mineral dusts remaining *in situ* produced granulomas throughout the experiment.

Saline. One injection of equal volume to that used for silica or the initial dose of carrageenan.

*Batch* 7.—Talc, Italian talc (Code 00000). 40 mg in 2 equal doses with an 8-week interval between doses.

All substances were autoclaved and suspended in physiological saline.

Each rat was allowed to live until death, unless it appeared to be distressed, when it was killed by chloroform exposure. A full necropsy examination was carried out on all rats, except for the control in Batch 7 that died only 2 days after asbestos injection. One rat in Batch 7 that received talc treatment was lost.

### RESULTS

The mean survival time, number of mesotheliomas, and time of first mesothelioma are given in Table II, and the distribution of survival times in the Figure.

TABLE II.—*Results*

| Batch | Supplement | Mean Survival (days) | No. of mesothelioma/ No. injected | Time from injection to first mesothelioma (days) |
|---|---|---|---|---|
| 1 | — | 706 | 4/12 | 535 |
| | BCG | 663 | 6/12 | 550 |
| 2 | — | 727 | 6/12 | 650 |
| | BCG | 693 | 3/12 | 495 |
| 3 | — | 667 | 5/12 | 587 |
| | BCG | 680 | 2/12 | 473 |
| 4 | — | 691 | 2/12 | 810 |
| | BCG | 714 | 1/12 | 832 |
| 5 | — | 652 | 2/12 | 579 |
| | BCG | 588 | 2/12 | 377 |
| Total | — | 688 | 19/60 | 535 |
| 1–5 | BCG | 668 | 14/60 | 377 |
| 6 | Saline | 657 | 14/32 | 399 |
| | Silica | 641 | 15/28 | 492 |
| | Carrageenan | 606 | 20/28 | 424 |
| 7 | — | 592 | 9/24 | 506 |
| | Talc | 604 | 7/23 | 455 |

In the group in Batch 6 injected with silica 8 months after the asbestos injection there were 3 rats which developed a lymphoma (Wagner & Wagner, 1972). All 3 of these also had a mesothelioma, 711, 712 and 823 days after the asbestos injection. One other rat, which did not have a mesothelioma and died 734 days after the asbestos injection, was noted as "? silica injection-site tumour".

The differences in mesothelioma incidence have been analysed by the conditional likelihood method of Cox (1972). This method estimates the relative incidence of tumours, after making the necessary allowance for mortality due to other causes, differences in survival time, and, in the case of the BCG effect, variation between Batches 1–5. All rats dying before the occurrence of the first mesothelioma in each batch are excluded from the analysis.

FIG.—Distribution of survival times and tumours according to treatment. □ 1 rat; ▩ 1 rat with mesothelioma.

The time schedule of the BCG treatment differed between batches, but there was no evidence of heterogeneity between Batches 1–5 in the size of the BCG effect. This could have been because the separate batches were small. Using the data from all the batches together, the mesothelioma incidence in the BCG-treated rats was 70% of the rate of the comparable group (Table III); this reduction could have been due to chance ($P = 0{\cdot}4$). In Batch 6

TABLE III.—*Analysis of supplementary treatments*

| Batch | Supplement | % Mesos | Relative meso incidence (95% limits in brackets) |
|---|---|---|---|
| 1–5 | — | 32 | 1·0 |
| | BCG | 23 | 0·7 (0·4–1·5) |
| 6 | Saline | 44 | 1·0 |
| | Silica | 54 | 1·1 (0·5–2·3) |
| | Carrageenan | 71 | 3·1 (1·5–6·5) |
| 7 | — | 39 | 1·0 |
| | Talc | 30 | 0·8 (0·3–2·3) |

the slight increase in the silica group was not significant. The mesothelioma incidence in the carrageenan group was 3·1 times that in the saline group ($P < 0·01$); the estimated relative effect was greater than the increase in the proportion of rats with mesotheliomas (71% *vs* 44%). This is because Cox's method allowed for the 50 days' reduction in average survival in the carrageenan group, compared with the saline group (Table II). In Batch 7, the slight reduction in the talc group could also have been due to chance.

### DISCUSSION

BCG therapy at the time the tumour usually develops obviously has little effect. Evans & Alexander (1972) have shown that macrophages taken from an animal sensitized to PPD, when reintroduced to PPD, will nonspecifically kill tumour cells. It is of importance in the future, therefore, to investigate whether the tumour rate could be altered if the first dose of BCG was given before the intrapleural crocidolite, followed by other doses at the time the tumour would develop.

The differences in the results found with silica, talc and carrageenan are interesting. Silica (O'Rourke *et al.*, 1978) has been shown to kill macrophages and not lymphocytes. Carrageenan (Allison *et al.*, 1966) is known to be cytotoxic towards macrophages, whereas there is no such evidence available for talc. Keller (1976) has stated that carrageenan gave an analogous or even more pronounced enhancement of tumour growth than silica. It has been suggested by Allison (1976) that silica may only temporarily deplete macrophages. Silica was given in only one dose in contrast to 3 doses of carrageenan, in which the first dose was later. According to Keller (1976) the enhancing effects of silica and carrageenan are "sharply circumscribed" and only when given at the same time as s.c. tumour cells is there a marked enhancement of tumour growth. The difference between silica and carrageenan in our experiment could then be accounted for by the different inoculation schedules, (carrageenan being perhaps given at a more appropriate time) and because carrageenan is, according to Keller, more potent in advancing tumour growth.

That carrageenan produced mesotheliomas is unlikely, as in the preliminary study it was found that the reaction caused by this material was transitory. Although there is no proof that there was no persistent reaction after the 3 injections, only fibrous dusts have been shown to produce mesotheliomas. Previous work has shown that silica and talc do not produce mesotheliomas after intrapleural injection (Wagner & Wagner, 1972; Wagner *et al.*, 1977).

If the work of Yung & Cudkowicz (1977) on rejection of foreign bone grafts is relevant to tumour rejection, the anti-complementary effect of carrageenan (which they have shown does not weaken rejection) will not be the effective factor in this instance. The depression of immune responses reported by Thompson *et al.* (1976) and Schwartz & Catanzaro (1973) may also be due to the antimacrophage effect of carrageenan.

However, in a recent evaluation of the effects of carrageenan on macrophages *in vitro*, Simon & Jones (1979) suggested that while macrophages contained carrageenan they remained viable and capable of phagocytosing carbon. Yung & Cudkowicz (1978) have proposed that carrageenan induces transition from the pre-suppressor to the suppressor state of a macrophage-like cell. Moreover, this causes the failure of generation of cytotoxic T lymphocytes and the generation instead of T suppressor cells. Perhaps the different indirect action of carrageenan on T lymphocytes may help to account for the discrepancy we find between carrageenan and silica.

### REFERENCES

ALLISON, A. C. (1976) Fluorescence microscopy of lymphocytes and mononuclear phagocytes and the use of silica to eliminate the latter. In *In vitro Methods in Cell-mediated and Tumour Immunity.* Eds Bloom & David. New York: Academic Press. p. 395.

ALLISON, A. C., HARINGTON, J. S. & BIRBECK, M. (1966) An examination of the cytotoxic effects of silica on macrophages. *J. Exp. Med.*, **124**, 141.

COX, D. R. (1972) Regression models and life tables. *J. R. Statist. Soc. B.*, **34**, 187.

EVANS. R. & ALEXANDER, P. (1972) Mechanism of immunologically specific killing of tumour cells by macrophages. *Nature*, **236**, 168.

KELLER, R. (1976) Promotion of tumour growth *in vivo* by antimacrophage agents. *J. Natl Cancer Inst.*, **57**, 1355.

O'ROURKE, E. J., HALSTEAD, S. B., ALLISON, A. C. & PLATTS-MILLS, T. A. E. (1978) Specific lethality of silica for human peripheral blood mononuclear phagocytes *in vitro*. *J. Immunol. Methods*, **19**, 137.

SCHWARTZ, H. J. & CATANZARO, P. J. (1973) The differential suppression of antigen, lymphokine and mitogen-induced delayed hypersensitivity-type reactions by carrageenan. *Int. Arch. Allergy Appl. Immunol.*, **44**, 409.

SIMON, L. & JONES, T. L. (1979) Re-evaluation of carrageenan cytotoxicity for macrophages. *J. Reticuloendothel. Soc.*, **25**, 133.

THOMPSON, A. W., WILSON, A. R., CRUICKSHANK, W. J. & HORNE, C. H. W. (1976) Evaluation of carrageenan as an immunosuppressive agent and mediator of intravascular coagulation. *Biomedicine*, **24**, 102.

TIMBRELL, V., GILSON, J. C. & WEBSTER, I. (1968) UICC standard reference samples of asbestos. *Int. J. Cancer*, **3**, 406.

WAGNER, J. C. & BERRY, G. (1969) Mesotheliomas in rats following inoculation with asbestos. *Br. J. Cancer*, **23**, 567.

WAGNER, J. C., BERRY, G. & TIMBRELL, V. (1973) Mesotheliomas in rats after inoculation with asbestos and other materials. *Br. J. Cancer*, **28**, 173.

WAGNER, J. C., BERRY, G., COOKE, T. J., HILL, R. J., POOLEY, F. D. & SKIDMORE, J. S. (1977) Animal experiments with talc. In *Inhaled particles IV*. Ed. Walton. Oxford: Pergamon. p. 647.

WAGNER, M. M. F. (1979) Thymectomy and asbestos-induced mesotheliomas in rats. *Br. J. Cancer*, **39**, 337.

WAGNER, M. M. F. & WAGNER, J. C. (1972) Lymphomas in the Wistar rat after intrapleural inoculation of silica. *J. Natl Cancer Inst.*, **49**, 81.

YUNG, Y. P. & CUDKOWICZ, G. (1977) Abrogation of resistance to foreign bone marrow grafts by carrageenans. II. Studies with the anti-macrophage agents, iota, kappa and lambda, carrageenans. *J. Immunol.*, **119**, 1310.

YUNG, Y. P. & CUDKOWICZ, G. (1978) Suppression of cytotoxic T lymphocytes by carrageenan-activated macrophage-like cells. *J. Immunol.*, **121**, 1990.